# CAD/CAM splint based on soft tissue 3D simulation for treatment of facial asymmetry

**DOI:** 10.1186/s40902-016-0050-8

**Published:** 2016-01-27

**Authors:** Kazuhiro Tominaga, Manabu Habu, Hiroki Tsurushima, Osamu Takahashi, Izumi Yoshioka

**Affiliations:** 1 Department of Science of Physical Functions, Division of Oral and Maxillofacial Surgery, Kyushu Dental University, Kitakyushu, Japan; 2 Department of Science of Physical Functions, Division of Oral Medicine, Kyushu Dental University, Kitakyushu, Japan

**Keywords:** Facial asymmetry, 3D simulation, Soft tissue, CAD/CAM, Orthognathic surgery, Horseshoe osteotomy, Positioning device

## Abstract

**Background:**

Most cases of facial asymmetry involve yaw deformity, and determination of the yaw correction level is very difficult.

**Methods:**

We use three-dimensional soft tissue simulation to determine the yaw correction level. This three-dimensional simulation is based on the addition of cephalometric prediction to gradual yaw correction. Optimal yaw correction is determined visually, and an intermediate splint is fabricated with computer-aided design and computer-aided manufacturing. Application of positioning devices and the performance of horseshoe osteotomy are advisable.

**Results:**

With this procedure, accurate repositioning of jaws was confirmed and patients obtained fairly good facial contour.

**Conclusions:**

This procedure is a promising method for a widespread, predictable treatment of facial asymmetry.

## Background

Management of facial asymmetry is very challenging. Treatment followed by two-dimensional (2D) analysis alone, such as cephalometric prediction, can achieve midline correction. However, it is insufficient for facial contour correction. Three-dimensional (3D) models and/or 3D simulation are often used to overcome this issue. However, these bone-level simulations also have some limitations. Most cases of asymmetry require correction of yaw, and changes in yaw have a significant influence on the facial contour. Evaluation of facial contour is not sufficient only from the frontal and lateral directions; it should also be performed from the oblique direction. Our strategy for facial asymmetry is as follows. Using soft tissue simulation, gradual changes in yaw are evaluated from various angles to determine the optimal correction of yaw. Based on the determined movement, an intermediate splint is made with computer-aided design and computer-aided manufacturing (CAD/CAM). We developed 3D positioning devices [1, 2] to maintain the original position of the mandible during surgery. Because treatment of asymmetry often requires upward and/or backward repositioning of the maxillary segment bilaterally or unilaterally, we developed a modified horseshoe osteotomy technique to avoid bony interference of the posterior part of the maxilla [3, 4]. This sequential process is explained through a case presentation.

## Methods

Our strategy for treatment of facial asymmetry is followed.

**Fig. 1:**
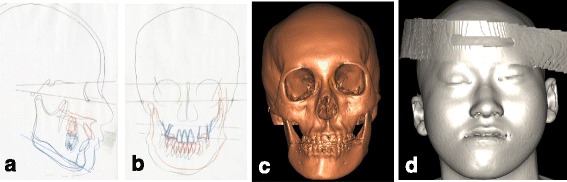
**a**, **b** Cephalometric prediction. **c**, **d** Presurgical volumetric representation with presurgical splint

**Fig. 2:**
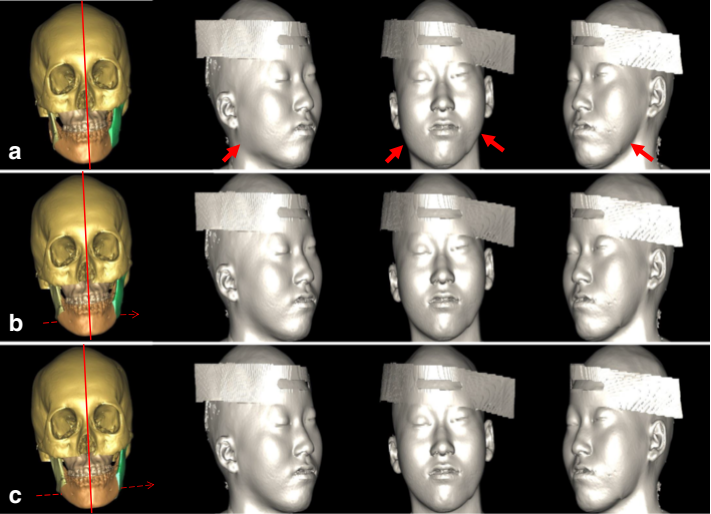
**a** Virtual simulation based on cephalometric prediction. Although midline correction was achieved (*red line*), the contour correction was inadequate (*arrows*). **b** The maxillomandibular complex was yawed, maintaining the anterior midline as a hinge, i.e., the posterior part of the complex was rotated toward the direction indicated by the *dotted arrow*. **c** More yawed than in (**b**). In this case, **b** was selected as the best reposition

**Fig. 3:**
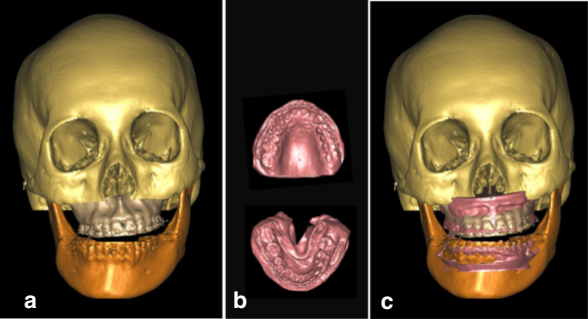
**a** Volumetric representations of dental arch plaster models scanned with a laser. **b** Virtual simulation of the maxillary reposition of Fig. 2b. The mandible is in the original position. **c** Virtual augmentation of (**a**) and (**b**). Fusion of the images can be automatically achieved by registration of three discriminated points of braces

**Fig. 4:**
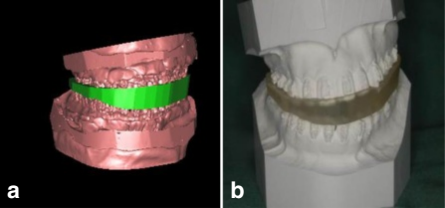
**a** Computer-aided designed intermediate splint. **b** Computer-aided manufactured splint

**Fig. 5:**
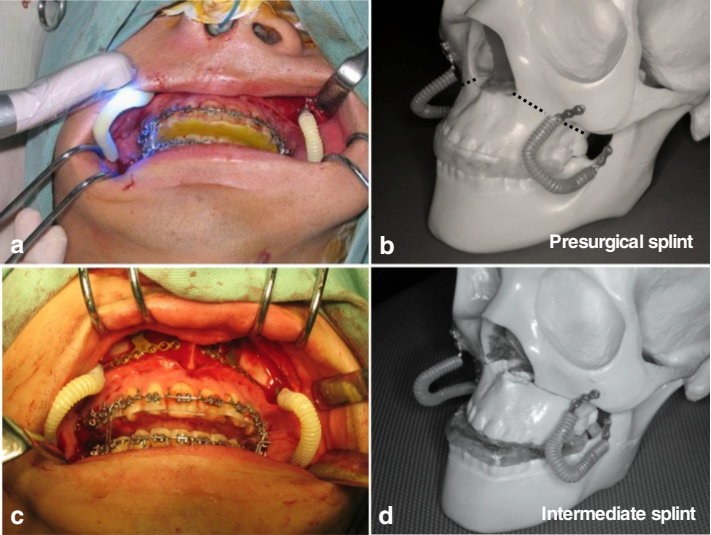
Three-dimensional positioning technique. **a**, **b** The positioning devices are used to record the original position of the mandible with the presurgical splint. Light curing resin in a flexible tube is hardened by irradiation with an LED light. **c**, **d** Repositioning of the maxillary segment using the intermediate splint after down fracture

1. Classic cephalometric prediction is performed based on the results of conventional 2D soft tissue and hard tissue analyses (Fig. 1a, b).
2. Computed tomography (CT) is performed with a presurgical splint that slightly opens the mandible from centric occlusion. The DICOM data of CT are converted into simulation software (SimPlant Pro OMS Ver. 13.0; Materialize, Leuven, Belgium) (Fig. 1c, d).
3. The metrical analysis results of cephalometric prediction are input into the simulation software, and 3D virtual prediction is performed (Fig. 2a). Midline correction can be achieved in many cases only by correction of the pitch and roll based on frontal and lateral cephalograms. Without yaw correction, however, a satisfactory facial contour cannot be achieved.
4. Gradual yaw correction while maintaining the anterior midline as a hinge is performed on soft tissue virtual simulation. Optimal yaw correction is determined visually not only from the frontal view but also from the oblique view (Fig. 2b, c). The facial contour gradually improves with correction of the yaw; at a certain critical point, however, the contour becomes worse. This critical point is the optimal point of yaw correction. However, multiple inspectors are required because this is an inspectional determination. The critical point is usually determined by one or more oral surgeons and orthodontists. The movement is determined from soft tissue-based simulation and is usually too complex to realize with facebow transfer and plaster model trimming because laboratory error is much larger.
5. An intermediate splint is produced by CAD/CAM. First, plaster models of the dental arches are laser-scanned (Fig. 3a). Using the virtual repositioning situation of the maxilla (the mandible is in an in situ position) (Fig. 3b), the digital data of the dental arches are fused to the bone data (Fig. 3c). Based on this intermaxillary relationship, an intermediate splint is produced by CAD/CAM (Fig. 4).
6. For proper repositioning of the maxilla using the intermediate splint, the mandible should be in exactly the same position as that on presurgical CT with the presurgical splint. A simple 3D positioning device that we developed [1, 2] is then used (Fig. 5).
7. Because maxillary deficiency is rarely involved in cases of facial asymmetry, most affected patients require backward and upward repositioning of the maxilla unilaterally or bilaterally. In these cases, complete removal of bony interference of the posterior part of the maxilla is mandatory. Application of the modified horseshoe osteotomy technique that we developed is effective for this purpose [3–5].

## Results

**Fig. 6:**
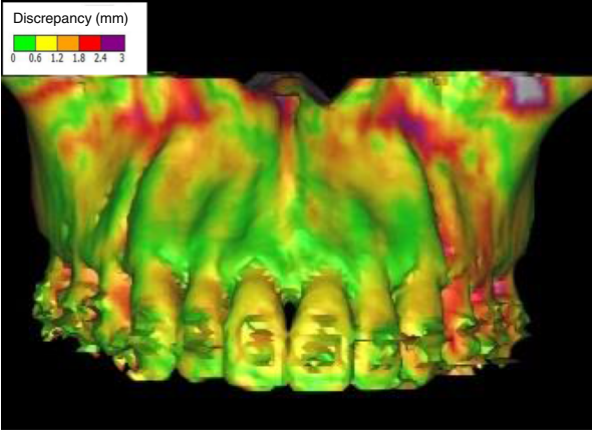
Visual superimposed volumetric image of hard tissue discrepancy between the virtual planning result and 3-month postoperative result. Discrepancies can be recognizable by colors. Good bony reproduction can be achieved

**Fig. 7:**
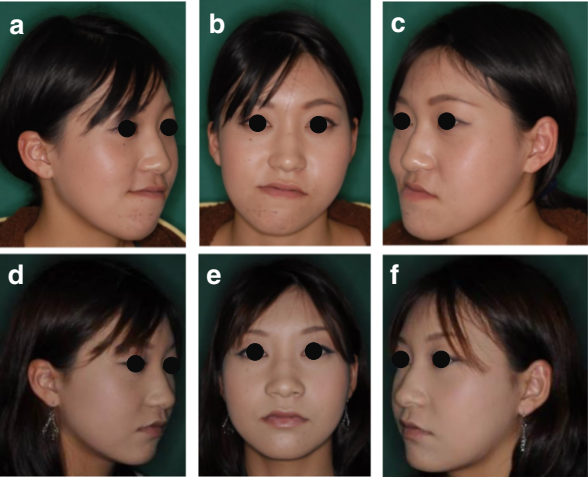
Preoperative (**a**–**c**) and postoperative (**d**–**f**) photographs. Oblique views are important to evaluate residual deformity

Some simulation software has recently furnished a function that provides a superimposed volumetric image of the discrepancy between the virtual planning result and the postoperative result. Our strategy ensures effective bony reproduction and better facial symmetry (Figs. 6 and 7).

## Discussion

In simple cases of jaw deformity without yaw correction, conventional cephalometric prediction works well. However, patients with yaw deformity require analyses with 3D models or 3D virtual simulation. In most cases of facial asymmetry, even if 3D analyses are performed, hard tissue simulation alone does not provide a satisfactory outcome. We therefore propose yaw correction based on soft tissue simulation to overcome this problem. Our major correction strategy is based on cephalometric analysis with an enormous amount of accumulated knowledge, and soft tissue virtual simulation covers the weak points. Although the accuracy of virtual soft tissue prediction does not reach the level of the actual use of postoperative prediction [6], soft tissue evaluation of virtual yaw correction might provide a better vector to obtain better facial contour. Further research is needed before virtual soft tissue simulation is used for soft tissue prediction.

Optimal reproduction of this simulation technique in the operative field is necessary to successfully use this procedure. European researchers invented interactive visualizing displays to reposition the maxilla into the simulated position [7]. However, they were unrealistic for routine orthognathic surgery and were extremely time-consuming and costly. Owing to recent improvement and popularization of 3D printers, various types of surgical stents with CAD/CAM for positioning of bone segments as well as osteotomy guides have been reported [6, 8, 9]. Although they require less time and cost, a certain amount of manpower and money are also necessary to design and manufacture these complex structures.

The CAD/CAM splint we used is custom-made by a manufacturer. The manufacturer also performs laser-scanning of plaster dental models. The operators must only perform virtual simulation and fusion between the jaw simulation and dental arch data. Our technique combines the CAD/CAM splint and a simple intraoperative positioning device, thus using the least cost and time to achieve the desired outcome. Additionally, it is possible to achieve upward and backward repositioning of the maxilla without bony interference; this is often needed in cases of facial asymmetry. The accuracy and reliability of the combination of the positioning device and horseshoe osteotomy have been reported elsewhere [10].

## Conclusions

Our strategy can be used anywhere that simulation software is available and is a promising method for a widespread, predictable treatment of facial asymmetry.

## Consent

Written informed consent was obtained from the patient for the publication of this report and any accompanying images.
